# Live to cheat another day: bacterial dormancy facilitates the social exploitation of β-lactamases

**DOI:** 10.1038/ismej.2015.154

**Published:** 2015-10-27

**Authors:** Frances Medaney, Tatiana Dimitriu, Richard J Ellis, Ben Raymond

**Affiliations:** 1 School of Biological Science, Royal Holloway University of London, Egham, UK; 2 Department of Life Sciences, Imperial College, Ascot, UK; 3 Specialist Scientific Support Department, Animal and Plant Health Agency, APHA Weybridge, Addlestone, UK

## Abstract

The breakdown of antibiotics by β-lactamases may be cooperative, since resistant cells can detoxify their environment and facilitate the growth of susceptible neighbours. However, previous studies of this phenomenon have used artificial bacterial vectors or engineered bacteria to increase the secretion of β-lactamases from cells. Here, we investigated whether a broad-spectrum β-lactamase gene carried by a naturally occurring plasmid (pCT) is cooperative under a range of conditions. In ordinary batch culture on solid media, there was little or no evidence that resistant bacteria could protect susceptible cells from ampicillin, although resistant colonies could locally detoxify this growth medium. However, when susceptible cells were inoculated at high densities, late-appearing phenotypically susceptible bacteria grew in the vicinity of resistant colonies. We infer that persisters, cells that have survived antibiotics by undergoing a period of dormancy, founded these satellite colonies. The number of persister colonies was positively correlated with the density of resistant colonies and increased as antibiotic concentrations decreased. We argue that detoxification can be cooperative under a limited range of conditions: if the toxins are bacteriostatic rather than bacteridical; or if susceptible cells invade communities after resistant bacteria; or if dormancy allows susceptible cells to avoid bactericides. Resistance and tolerance were previously thought to be independent solutions for surviving antibiotics. Here, we show that these are interacting strategies: the presence of bacteria adopting one solution can have substantial effects on the fitness of their neighbours.

A cooperative trait is a behaviour by one individual that can benefit another (West *et al.*, 2006). In bacteria, cooperative traits often come in the form of ‘public goods' released into the environment and available to all. Many virulence factors, including siderophore production, Cry proteins and quorum-regulated traits are cooperative (West and Buckling, 2003; Diggle *et al.*, 2007; Sandoz *et al.,* 2007; Raymond *et al.*, 2012; Zhou *et al.*, 2014). Antibiotic resistance conferred by the enzymatic breakdown of drugs is potentially a cooperative trait as it can detoxify the environment for all cells, and the production of β-lactamases, which cleave and deactivate penicillins, is often cited as a social trait in bacteria (West *et al.*, 2006; Diggle *et al.,* 2007; Brown *et al.*, 2009). Clinical studies have suggested that protective clearance is mediated by the release of β-lactamase enzymes into the environment by producing cells (Brook, 2004), and packaging of β-lactamases into extracellular vesicles has been demonstrated in *Pseudomonas aeruginosa* (Ciofu *et al.*, 2000). Secretion of enzymes could increase the area of antibiotic clearance, benefiting all cells in a local population.

The phenomenon of protective clearance of antibiotics by resistant cells is commonly seen by microbiologists in the presence of ‘satellite' colonies on transformation plates (Figure 1a). These non-resistant colonies are able to grow on ampicillin plates where resistant colonies are already established. A plausible hypothesis that explains this phenomenon is that resistant transformants clear the antibiotic from their immediate vicinity, creating an antibiotic-free space where susceptible ‘satellite' colonies can then grow. Previous studies have demonstrated the survival of antibiotic-sensitive *Escherichia coli* and *Salmonella sp.* in the presence of resistant strains at high concentrations of antibiotic *in vitro* (Dugatkin *et al.*, 2005; Clark *et al.*, 2009; Perlin *et al.*, 2009). Cross-species protection of susceptible bacteria by β-lactamase producers has also been seen *in vivo* (Tacking, 1954; Hackman and Wilkins, 1975; Brook *et al.*, 1983), suggesting that the benefits of β-lactamase enzymes may spread to entire communities.

Although social evolution theory has done much to alter and improve modern microbial ecology, there is some justification for being cautious about claiming whether specific traits are cooperative or not. A recent controversy has highlighted two important points when studying cooperation: first, we need to demonstrate that behaviours have real fitness benefits for populations, and second that it is desirable to study social evolution with realistic models (Zhang, 2004; Ghoul *et al.*, 2014), something we have endeavoured to do in previous studies (Raymond *et al.*, 2012; Zhou *et al.*, 2014). For example, demonstrating that a microbial product is secreted is not sufficient evidence for cooperation; spatial structure, for example, can prevent secreted products from being publicly available (Raymond and Bonsall, 2013; Zhou *et al.*, 2014), or metabolic products may not be beneficial in all contexts (Zhang and Rainey, 2013; Ghoul *et al.*, 2014). Conversely, secretion may not be necessary for cooperation in the case of detoxification. Active removal of toxins and the ensuing diffusion gradients or lowered concentration of toxin may be all that is required to protect susceptible bacteria (Lenski and Hattingh, 1986).

There are some grounds for being sceptical about any claim that β-lactamases are generally cooperative. Previous experiments have used model bacteria with altered sites of expression and potentially increased secretion (Dugatkin *et al.*, 2005; Perlin *et al.*, 2009) and these data might not reflect social interactions in more natural conditions. It is important therefore to consider whether antibiotic resistance genes in the more realistic context of naturally-occurring plasmids can lead to cooperative detoxification of antibiotics. An additional consideration is whether the antibiotic in question is bacteriostatic or bactericidal. When β-lactams are bacteriostatic, that is, if they suspend growth but do not rapidly kill bacteria, then the potential for social interactions may be increased as susceptible bacteria may survive until detoxification by neighbours can reduce concentrations of antibiotics to below inhibitory doses. However, β-lactam antibiotics can be bactericidal, that is, rapidly lethal to bacteria, under a range of conditions (Rolinson *et al.*, 1977; Cozens *et al.*, 1986). Any bactericidal activity is expected to substantially restrict the conditions for coexistence and social exploitation to a narrow range that may depend on initial dosage, as well as the frequency of resistant and susceptible bacteria (Lenski and Hattingh, 1986; Levin, 1988).

However, bacteria do have mechanisms that enable them to escape or tolerate the effects of bactericidal antibiotics, one being a ‘persister' state in which dormant cells can survive exposure to antibiotics (Lewis, 2010). Persister cells were identified early in the clinical life of penicillin (Bigger, 1944), but a recent resurgence in interest has been fuelled by a wider appreciation of their clinical importance, especially in the light of the current antibiotic resistance crisis (Lewis, 2007). Persister cells are natural variants present at low frequency in the bacterial population (Lewis, 2010). The phenotypic switch between persistence and active growth appears to occur at random, although it is affected by growth phase (Balaban, 2004). The presence of persisters in biofilms is thought to contribute to increased antimicrobial tolerance and the maintenance of chronic infections (Brooun *et al.*, 2000; Lewis, 2001; Harrison *et al.*, 2005). Since satellite colonies on ampicillin plates are characterized by a delayed growth pattern, appearing after 24–72 h of cultures, and emerge as rare individuals from a high density of bacteria, they have many of the characteristics of cells that have passed through a persister state. We therefore hypothesised that phenotypically susceptible bacteria might only rarely be able to benefit from detoxification by others while persisters may be more likely to exploit the β-lactamases of their neighbours.

The primary aim of this work was to examine cooperative β-lactam resistance using a naturally occurring resistance plasmid, pCT, (Cottell *et al.*, 2011) and to investigate both the environmental and demographic conditions under which cooperative resistance occurs. In line with social evolution theory, we expected that ‘cheating' or exploitation of detoxification by susceptible cells should increase with the density and frequency of resistant bacteria (Ross-Gillespie *et al.*, 2007, 2009; Raymond *et al.*, 2012). To this end, competition experiments were conducted between the pCT-carrying strain and an otherwise isogenic plasmid-free *E. coli* under a variety of conditions. The results of these experiments showed little or no cooperative resistance, in other words phenotypically susceptible bacteria did not tend to have an increased ability to survive antibiotics in the presence of resistant cells. We then tested whether dormancy has a role in the ability of *E. coli* to exploit the β-lactamase activity of neighbouring cells.

## Materials and methods

### Strains and culture techniques

Our standard culture conditions used either 5 ml Luria Bertani (LB) broth (Fisher Scientific UK Ltd., Loughborough, UK), or agar plates with LB broth and 2% (w/v) agar (Agar Bacteriological No. 1, Oxoid, Basingstoke, UK). Broth and plates were supplemented with antibiotics as required; pCT plasmid-carrying strains were maintained on plates containing either ampicillin (100 μg ml^−1^, sodium salt, Sigma-Aldrich, Gillingham, UK) or cefotaxime (8 μg ml^−1^, sodium salt, Melford Laboratories, Ipswich, UK).

Initial detoxification bioassays used *E. coli* DH10B+pCT as the resistant strain, while all subsequent competition experiments used *E. coli* K-12 MG1655. We created readily distinguishable *E. coli* K-12 MG1655 by generating a *lac* null knockout mutant via disruption of chromosomal *lacZYA* genes (nucleotides 360 842–365 662). This produces a *Δlac* mutant with a white phenotype in the presence of X-Gal and IPTG, while wild-type bacteria are blue on this media. Mutants were produced using the Xercise protocol, as described by Bloor and Cranenburgh (2006). Diagnostic PCR was used to assess the insertion and subsequent deletion of the dif-CAT-dif fragment into MG1655. Diagnostic PCR was conducted using Taq (Qiagen, Manchester, UK) with an annealing temperature of 55 °C and using the primer F Lac diag (5′-ACGGAAAGAGTAACGTTGGGTGC-3′) and R Lac diag (5′-GCGCCATTACCGAGTCCGGG-3′). Deletion primers for MG1655 *Δlac* were taken from Datsenko and Wanner (2000). The fitness cost of the *lacZYA* knockout was assessed with competition on plates. We introduced the resistance plasmid pCT in the wild-type *E. coli* K-12 MG1655 via electroporation. The *ΔlacZYA* MG1655 white mutant was used as our susceptible strain.

### Detoxification bioassay

**Figure 1:**
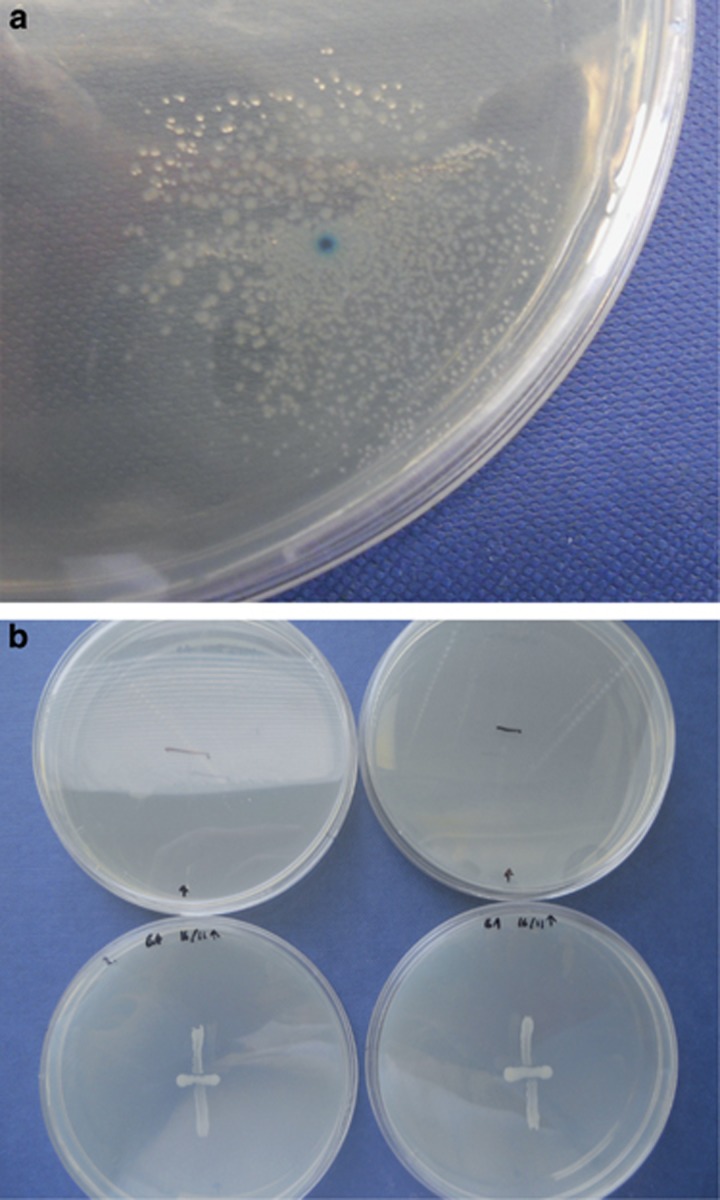
(**a**) Satellite colonies around a successful pUC19 transformant on ampicillin agar. Transformation of *E. coli* DH10B with pUC19 confers resistance to ampicillin and also restores the lac operon, resulting in a blue colony on agar containing X-Gal and IPTG. The successful transformants appeared after ~16 h, white susceptible colonies appeared after >24 h incubation and can be seen growing around the resistant transformants. (**b**) Antibiotic clearance bioassay. Susceptible colonies grow on ampicillin plates in the presence of resistant colonies (bottom row), but not alone (top row). Resistant colonies were incubated for 48 h on 100 μg ml^−1^ ampicillin plates before the addition of susceptible colonies. Susceptible strains can grow in the region around the resistant colony, but do not grow outside the central zone or in the absence of a resistant colony (top row). These assays were repeated seven times.

A simple bioassay was used to assess whether pCT-carrying bacteria were capable of clearing β-lactam antibiotics from solid media. A single resistant colony was streaked onto LB agar containing 100 μg ml^−1^ ampicillin and incubated at 37 °C for 48 h (Figure 1). Single colonies of susceptible *E. coli* were then streaked from the central resistant colony to the edge of the plates and onto plates with no resistant colony (Figure 1). Plates were incubated at 37 °C overnight and growth measured from the central colony outwards. In a further assay, the central resistant colony was killed using a chloroform soaked filter disk, applied to the central colony for 10 min and then removed and allowed to dry before addition of the susceptible strain. Experiments were repeated over a range of ampicillin concentrations.

### Competition experiments

Competition experiments were conducted on LB agar plates containing X-Gal (0.02 mg ml^−1^) and IPTG (0.1 mM). We chose a range of antibiotic doses that would bracket the minimal inhibitory concentrations of ampicillin in the susceptible bacteria (4 μg ml^−1^) and cefotaxime (0.06 μg ml^−1^). OD_600_ of overnight (16 h) cultures of competing strains (susceptible and resistant) was measured and cultures diluted to approximately equal cell density with 0.85% (w/v) saline. Cultures were then mixed at ratios of 1:10, 3:10, 50:50 and 9:10 of resistant to susceptible cells and diluted with saline to give an approximate cell density of 1 colony-forming unit per μl (CFU μl^−1^). To confirm the initial ratio of cells, mixed cultures were plated onto LB agar+X-Gal and IPTG and incubated overnight at 37 °C, and blue and white colonies counted. In all experiments, 100 μl mixture or susceptible-only control was spread onto plates and incubated at 37 °C for 72 h. Colonies were then harvested by adding 5 ml saline to the plate, loosening colonies with a sterile spreader and homogenising with a pipette. Harvested colonies were serially diluted and plated onto LB agar+X-Gal and IPTG, with and without ampicillin. Plates were incubated at 37 °C overnight, and colonies counted to determine final ratios and relative fitness of the susceptible (MG1655 Δ*lacZYA*) strain. The overall design of the competition experiments is set out in Supplementary Figure S1. Three experiments were conducted, in which we manipulated (i) the initial proportion of resistant cells (approximately 0.1, 0.3 0.5, 0.9); (ii) the antibiotic (cefotaxime, rather than ampicillin) or (iii) the total density of bacteria per plate.

### Persister competition experiments

To increase numbers of persisters to detectable levels, a large volume of susceptible culture was used to inoculate competition plates. An 8-h starter culture in 5 ml LB broth was used to initiate 300 ml overnight culture by 1:1000 dilution. The overnight culture was centrifuged at 4000 *g* for 10 min, resuspended in 6 ml 0.85% saline and diluted 1:10 (no dilution was used in the varying dose experiment). Mixtures were made 50:50 with an overnight culture of resistant cells. Initial counts and competition plates were set up as described above. After 24 h, competition plates were moved to room temperature incubation (approximately 21 °C) for up to 3 weeks. Blue and white colonies were counted after 24 h, and then periodically. After 3 weeks, incubation plates were photographed and potential persister colonies screened. We defined persister colonies as being composed of phenotypically susceptible bacteria (that is, white) that appeared as strong colonies after a period of no growth (24 h after initial plating). We conducted two experiments that varied in (i) the initial number of resistant colonies per plate and (ii) the antibiotic dose.

We confirmed that late-appearing white colonies were phenotypically antibiotic susceptible, and therefore persisters, by picking a subset of colonies onto LB agar with and without ampicillin (at 100 μg ml^−1^), and by minimum inhibitory concentration (MIC) assays. We also tested whether putative persisters had acquired the pCT plasmid by transformation or conjugation by screening for the presence of pCT using primers designed to amplify the *trbA* plasmid-specific conjugation gene (FtrbA diag 5′-CGGCATCCAGGCAGGCATCA-3′ and RtrbA diag 5′-TTCAGCCCTGCCCGGTCATT-3′).

### Data analysis

The relative fitness of susceptible cells in competition experiments was calculated as described by Lenski (1988); also see Dahlberg and Chao (2003), and is given by the equation:


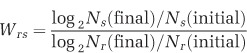


where *W*_*rs*_ is the fitness of strain *s* (susceptible) relative to strain *r* (resistant) and *N* is the cell density at the start and end of the experiment. Initial cell density was derived from the mean susceptible and resistant counts (CFU ml^−1^) in the antibiotic-free control plates in each experiment. It is not possible to calculate relative fitness when there is no detectable growth; therefore, all final cell counts were transformed by addition of the minimal detectable value, calculated as the ratio of the minimum possible CFU count over the maximum possible CFU count.

Relative fitness of susceptible bacteria and counts of persister colonies were analysed using generalised linear modelling, conducted in *R v3.02* (R Core Team, 2013). Since the responses of bacterial fitness to antibiotic were non-linear, we fitted log-transformed concentrations as a factor rather than as a covariate.

## Results

### Detoxification bioassay demonstrates protective clearance by resistant bacteria

This bioassay provides a clear visualisation of the resistant strain's ability to facilitate growth of susceptible bacteria (Figure 1b, Supplementary Figure S2). The sensitive DH10B strain only grows on ampicillin plates in the region around the resistant DH10B+ pCT colony, despite being inoculated to the edges of the plate (bottom row), and does not grow at all in the absence of resistant colonies (top row). Similar results were obtained when the central colonies were killed with chloroform, indicating that susceptible growth is not due to some interaction (such as conjugation) between resistant and susceptible strains (data not shown). Further experiments with *E. coli* K-12 MG1655 showed that the extent of detoxification is dependent on ampicillin dose (Supplementary Figure S2).

### Fitness costs of plasmid carriage and of lacZ marker

**Figure 2:**
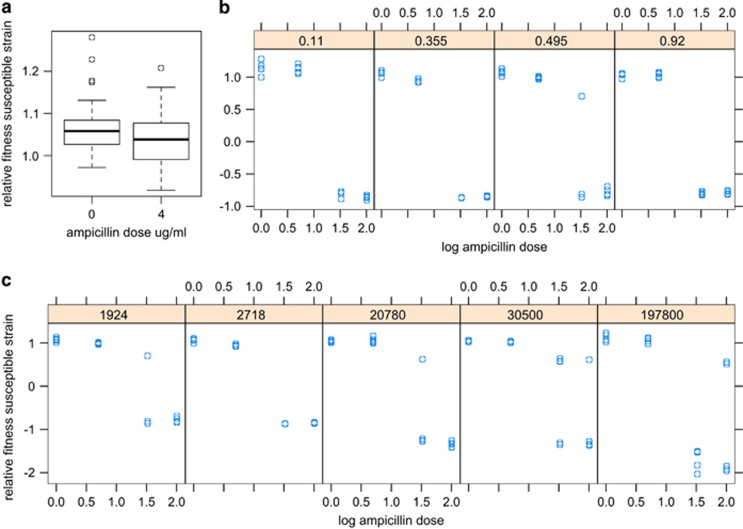
Relative fitness of susceptible bacteria in mixed populations at different antibiotic doses. (**a**) The fitness cost of plasmid carriage in the absence of ampicillin and at a sub-inhibitory dose. (**b**) At varying initial proportions of resistant bacteria, proportions are indicated in banners above panels. (**c**) At varying initial cell density on plates, where banner labels indicate CFU ml^−1^. Relative fitness of susceptible cells was calculated as described by Lenski (1988), a value of >1 indicates that susceptible bacteria higher relative fitness than the resistant competitor, and a value of <1 indicates lower relative fitness. Points are raw data from *n*=5 replicates per treatment.

We examined the relative fitness of susceptible bacteria at low doses of antibiotic (0 and 4 μg ml^−1^) in all competition experiments in order to assess the fitness burden of the carriage of pCT. This analysis was complicated by the fact that both cell density and frequency of plasmid carriage had significant effects on fitness at these doses (density—*F*_1,66_=4.64, *P*=0.035; frequency—*F*_1,67_=6.69, *P*=0.012). However, after taking these factors into account, the relative fitness of susceptible cells was estimated at 1.06 (s.e. 0.020; 95% confidence limits, 1.028–1.099; Figure 2a) for a ratio of 50:50 resistant and susceptible cells, indicating a small but significant cost of plasmid carriage. The fitness of susceptible bacteria decreased significantly as we increased the dose of ampicillin to 4 μg ml^−1^, a value below the MIC (*post hoc* test, estimated difference=−0.03, *t*=−2.213*, P*=0.03; Figure 2b). Preliminary experiments showed that MG1655 has very slightly lower fitness on agar plates than the white mutant MG1655 Δ*lacZYA* in the absence of antibiotic (mean relative fitness: 0.978, 95% confidence limits: 0.96–0.99).

### Relative fitness of susceptible bacteria across a range of antibiotic doses and densities of resistant cells

In all competition experiments on ampicillin, the relative fitness of susceptible bacteria declines dramatically at inhibitory doses (32 and 100 μg ml^−1^), with fitness values close to their minimum possible values in nearly all cases (Figure 2) (*F*_3,133_=232, *P*≪0.0001). This result was highly repeatable indicating that susceptible cells do not typically benefit from the presence of resistant cells at inhibitory doses of antibiotic, in contrast to the results of the detoxification bioassay (Figure 1b). The same pattern was observed in cefotaxime experiments, since the presence of resistant bacteria never allowed susceptible cells to survive and grow at doses of 0.064 μg ml^−1^ or above (data not shown).

In the susceptible-only single strain controls, there was no growth in the presence of inhibitory ampicillin doses. In a variant of this mixed strain assay in which doses were increased incrementally between 4 and 30 μg ml^−1^, growth of susceptible bacteria was only seen at 4 μg ml^−1^. We expected that an increased initial proportion of resistant bacteria might increase the fitness of susceptible bacteria, since more resistant bacteria should translate to more rapid detoxification of antibiotic. However, the initial proportion of resistant cells did not significantly affect relative fitness (*F*_1,131_=0.0159, *P*=0.90, Figure 2b) when we considered fitness at low and high doses of antibiotic.

Similarly, we expected that susceptible bacteria might be better able to survive when we increased the initial densities of all bacteria on plates. Here, we found that the density of colonies used to inoculate each plate had a small positive effect on relative fitness (*F*_1,132_=4.79, *P*=0.030, Figure 2c); although this effect interacted with antibiotic dose (*F*_3,129_=5.47, *P*=0.0014), and was weakest at 32 μg ml^−1^ (*post hoc t* test, effect=−5.28 × 10^−6^, s.e.=1.5 × 10^−6^, *t*=−3.43*, P*=0.00083). Notably, some isolated susceptible colonies were detected on plates with 32 μg ml^−1^ and 100 μg ml^−1^ of antibiotic in the presence of resistant bacteria, and this typically occurred at higher densities. These isolated colonies produced fitness values that are less than 1 but greater than zero. Figure 2 shows that these phenotypically susceptible bacteria were not consistently observed in all replicates within a particular dose. However, these data suggest that higher cell densities may facilitate some susceptible survival at inhibitory antibiotic doses, although this level of survival was low. An additional analysis, which used a Poisson-based model, and therefore could take into account the rare occurrence of susceptible colonies indicated that counts of susceptible colonies at inhibitory doses significantly increased with the log of the number of resistant bacteria at inoculation (*F*_1,75_=5.27, *P*=0.047, glm with quasipoisson errors; counts were observed after competition experiments were plated out at a standard dilution of 10^−6^).

### β-Lactam resistant bacteria facilitate growth of persisters

Here, we tested the hypothesis that susceptible bacteria can benefit more readily from the β-lactamase activity of their neighbours by undergoing a period of dormancy. In these experiments we used high population densities of susceptible bacteria, in order to increase the frequency of persisters to detectable levels. We monitored bacterial populations on plates for up to 3 weeks after initial exposure to antibiotics; late-appearing white colonies were interpreted as being founded by cells that had passed through a period of dormancy. Our screens of a subset of white putative persister colonies confirmed that they could not grow at ampicillin doses of 100 μg ml^−1^; that MICs were indistinguishable from our original susceptible strain and we could not amplify pCT-specific amplicons using the PCR.

**Figure 3:**
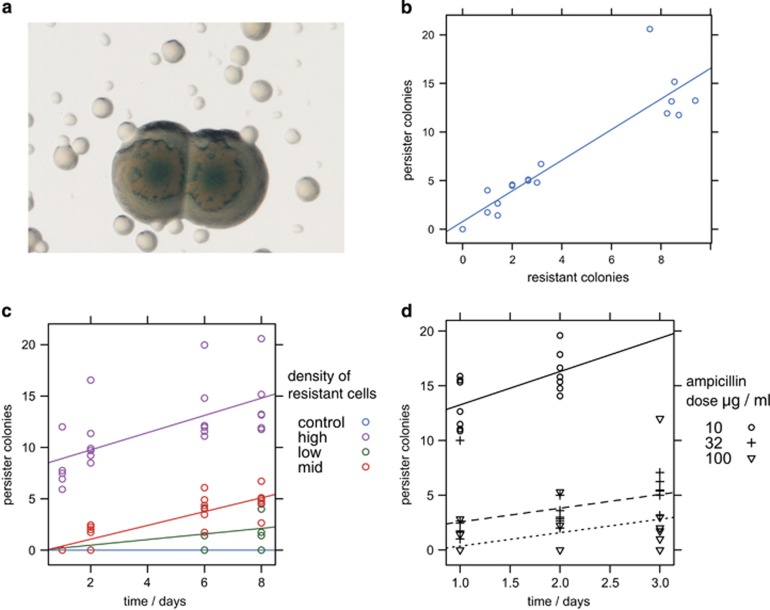
The relationship between persisters and the presence of resistant colonies. (**a**) × 3.0 magnification image showing resistant colonies (blue) and persister colonies (white) on LB agar 100 μg ml^−1^ ampicillin+X-Gal and IPTG. Resistant colonies appeared after overnight incubation at 37 °C. Persister colonies appeared after a further 24 h of incubation. (**b**) The number of persister colonies increases with the number of resistant colonies, data are numbers of persister and resistant colonies per plate after 8 days of incubation (both sqrt transformed). *R*^2^=0.8384. (**c**) Persister colonies appear more rapidly on plates containing higher number of resistant colonies (*n*=6 replicates). (**d**) Higher numbers of persister colonies were observed at lower doses of ampicillin (*n*=6 replicates).

At high concentrations of susceptibles (3.5 × 10^8^), late-appearing white susceptible colonies were observed close to blue resistant colonies even at the highest ampicillin dose, 100 μg ml^−1^ (Figure 3a). At ampicillin doses of 100 μg ml^−1^ these susceptible colonies were not seen in the control plates in the absence of resistant bacteria, confirming that this ability to re-enter active growth was dependent on a social interaction with their neighbours. Persister colonies did appear on control plates at 32 μg ml^−1^ when plates were incubated for 5 days at 21 °C.

The number of these susceptible persister colonies increases with the number of resistant colonies present (Figure 3b; *R*^2^=0.8384, *F*_1,16_=87.907, *P*<0.0001) and over time, as shown by the highly significant resistant colonies by time interaction term (Figure 3c; *F*_1,92_= 17.85, *P<*0.001). The number of persister colonies decreased with increasing antibiotic dose (Figure 3d, *F*_1,50_=55.62, *P*<0.001), and this experiment also shows a significant dose by resistant colony interaction term (*F*_1,50_= 6.780, *P*=0.0121). The key effects of dose, and the linear relationship between persisters and resistant cells, could be repeated when experiments were conducted at 37 °C (Supplementary Figure S3). All these data are consistent with the hypothesis that resistant cells are breaking down the antibiotic in their vicinity, facilitating the successful breaking of dormancy by phenotypically susceptible bacteria: resistant cells and lower antibiotic concentrations will lead to a more rapid reduction in antibiotic concentration and higher survival rates as cells cease being dormant.

## Discussion

In this study, bacterial cells that are susceptible to β-lactam antibiotics were only able to benefit from the protective clearance of those antibiotics by resistant cells under very specific conditions. We posit that a sub-population of these susceptible cells are ‘persisters': natural variants present at low frequency in the susceptible population, which survive high antibiotic concentration by dormancy (Lewis, 2010). Very high densities of susceptible bacteria in these experiments increased the numbers of persisters to a detectable level. Once the level of antibiotic is sufficiently reduced by the resistant cells, colonies founded by persister cells begin to appear. This explains both the lag before susceptibles appear, and the increase in numbers of persisters over time, as they switch back to metabolic activity at random (Balaban, 2004). Persister cells survive the initial high concentration of antibiotic by remaining dormant, but crucially their ability to grow is a social trait as it is dependent on the frequency and density of neighbouring β-lactamase producers. Persistence, therefore, facilitates cheating and the exploitation of antibiotic-free space provided by the resistant cells.

The observation that there is no social benefit for the susceptible strain except in the presence of persisters contrasts with previous work demonstrating protective clearance of antibiotics by resistant strains (Dugatkin *et al.*, 2005; Clark *et al.*, 2009; Perlin *et al.*, 2009; Yurtsev *et al.*, 2013). One possible explanation for this difference is that different strains and experimental set-ups may produce altered antibiotic tolerance. In these experiments, we show that any dose above the MIC is bactericidal to all growing *E. coli.* The bactericidal action of β-lactams is known to be variable and dependent on the specific drug, the strain, growth rate and stage, and the nutrient availability (Hobby *et al.*, 1942; Rolinson *et al.*, 1977; Cozens *et al.*, 1986; Tuomanen *et al.*, 1986). For example, the bactericidal effect of cefotaxime is similar to that of other β-lactams; however, cefotaxime has been shown to have remarkable potency against Enterobacteriaceae and to be bactericidal at doses at or close the MIC (Jones and Thornsberry, 1982; Neu, 1982). It is therefore possible that the experimental design of the earlier papers facilitated susceptible tolerance of doses above MIC, either through strain selection or through some element of the growth conditions, producing a bacteriostatic response to ampicillin rather than a bactericidal effect. This tolerance effect has been seen for mercury resistance, where a threshold exists above which susceptible cells are inhibited but not killed (Ellis *et al.*, 2007).

Perhaps a more likely explanation is that the part-batch, part-chemostat conditions used in these previous studies facilitate more rapid and generalised clearance of antibiotic than the strict batch culture method employed in this study. The model used by Dugatkin *et al.* (2005) and Perlin *et al.* (2009) allows build up of β-lactamases in one half of the chamber, and periodically replenishes the antibiotic in the other half. This build up of enzyme facilitates rapid breakdown of the antibiotic, similar to the earlier chemostat models in which coexistence occurs only when susceptible cells are added to an already established resistant culture (Lenski and Hattingh, 1986; Lenski, 1988). In a subsequent study, Dugatkin *et al.* recognise that enzyme accumulation may be a contributory factor (Clark *et al.*, 2009). The homogenous broth culture environment may facilitate more rapid breakdown of antibiotic simply by bringing more enzymes into contact with their substrate, as the law of mass action states. In addition, passive extracellular release of β-lactamases from the Gram-negative periplasm or ‘leakage' (Livermore, 1995) will have a greater impact in broth culture than in the solid batch culture environment. In this study, any exogenous enzyme would diffuse slowly through the agar, reinforcing the spatial structure of the environment, rather than clearing substantially more antibiotic. Both Clark *et al.* (2009) and Perlin *et al.* (2009) find substantially more extracellular β-lactamase in cultures where protective clearance is seen.

Dugatkin *et al.* (2005); Perlin *et al.* (2009) and Clark *et al.* (2009) all draw a distinction between ‘self-limited' and ‘shared' resistance, based on the localisation of the enzyme, enzymes in the shared resistance system being localised to the outer membrane. The results of this study question this differentiation, as no modifications to pCT have been made to alter the enzyme localisation, which is naturally largely periplasmic (Livermore, 1995). Yurtsev *et al.* (2013) point out that the site of antibiotic action determines the degree to which the resistant cells benefit from the public good, but that this does not affect the overall resistant-susceptible dynamic, which is determined by the rate of degradation alone. More β-lactamase was found in the supernatant of the ‘shared' resistance strain, due to breaking off of the enzyme from the cells outer membrane (Clark *et al.*, 2009), confirming the ‘altruistic' nature of resistance compared with the ‘self' resistance strain. However, this breaking off from the cell appears to incur a fitness cost for the ‘shared' resistance strain. This fact, coupled with free β-lactamase increasing the rate of degradation, could explain the survival of the susceptibles, rather than the enzyme localisation.

In this study, the degradation of antibiotic in culture was not fast enough to protect the majority of antibiotic susceptible cells: there was a time-lag before cooperative detoxification could be exploited. This impact of history on exploitation has implications for how we might theoretically describe the social biology of detoxification. Detoxification is typically considered as a snowdrift game, in which co-operators reap some direct benefit from their actions but in which there is also some element of a shared indirect benefit (Doebeli and Hauert, 2005). In these games it is best to cooperate when all your neighbours are defecting, and these interactions can promote stable co-existence of co-operators and defectors via negative frequency dependence (Doebeli and Hauert, 2005; Brown and Taddei, 2007). A simple assumption of these models is that the benefit of cooperation is a function of the frequency of co-operators. These models have been extended to incorporate historical effects, such as durable public goods, in this case the benefits of cooperation depend upon current and past abundance of cooperators (Brown and Taddei, 2007). Previous models of β-lactamase-based detoxification in broth have found that the dynamics can be summarised in terms of the concentration of antibiotics and the cell density (Yurtsev *et al.*, 2013). However, in order to describe the historical effects in this system, theory would also have to capture the density and mortality rate of susceptible bacteria as well as the current and past abundance of resistant cells.

The clinical implications of this work depend on whether detoxification of β-lactams in hosts resembles that in liquid media (widespread cheating) or on solid media (limited cheating). Arguably, colonies on solid agar are more akin to host-associated biofilms, which are also likely to contain persisters (Stewart and Franklin, 2008). In broth susceptible bacteria have strong competitive interactions with resistant cells leading to an equilibrium of 20–30% resistant cells at doses of 100 μg ml^−1^ ampicillin (Yurtsev *et al.*, 2013). This is potentially good news for resistance management if cheating means lactam antibiotics only weakly select for resistance. However, since this cheating depends on the public availability of β-lactamases there is a counter-intuitive outcome that the use of β-lactamase inhibitors can, at some doses, increase the frequency of resistant cells by removing their public services from extracellular space (Yurtsev *et al.*, 2013). In contrast, if β-lactamase detoxification in hosts is like that in solid media, then selection for resistance will be more straightforward, with only a very small fraction of persistent susceptible cells being able to survive antibiotic therapy. Effective therapeutic control of persisters is an ongoing challenge, but complex communities of bacteria with a mixture of tolerance and resistance strategies may require complex treatment with combination therapy. Combinations have the advantage of reducing the probability of bacteria of spontaneous mutations to multiple drugs (Worthington and Melander, 2013), but also, as in the treatment of tuberculosis, enable the control of bacteria with different growth rates, physiologies and susceptibilities (Mitchison, 1985).

Theory, and this work, suggests that the conditions for the coexistence of antibiotic resistant and susceptible bacteria are quite narrow: the initial antibiotic concentration must not exceed the lethal dose, and the rates of degradation and antibiotic influx must not allow the dose to increase (Lenski and Hattingh, 1986). Experiments with mercury resistance confirm that frequency-dependent coexistence of resistant and susceptible genotypes is strongly dose dependent (Ellis *et al.*, 2007). In this study, using batch culture, no dose greater than the MIC could be found that consistently allowed the coexistence of resistant and susceptible bacteria, unless the susceptibles made the phenotypic switch to persistence or resistant bacteria detoxified media before colonisation by a susceptible strain.
