# Supplementary material for: Live to cheat another day: bacterial dormancy facilitates the social exploitation of β-lactamases
Source: ISME J. 2015 Oct 27;10(3):778–87. doi: 10.1038/ismej.2015.154 (PMC4817691; doi:10.1038/ismej.2015.154)

Supplementary information for Medaney et al. “Live to cheat another day: bacterial dormancy facilitates the social exploitation of β-lactamases.”


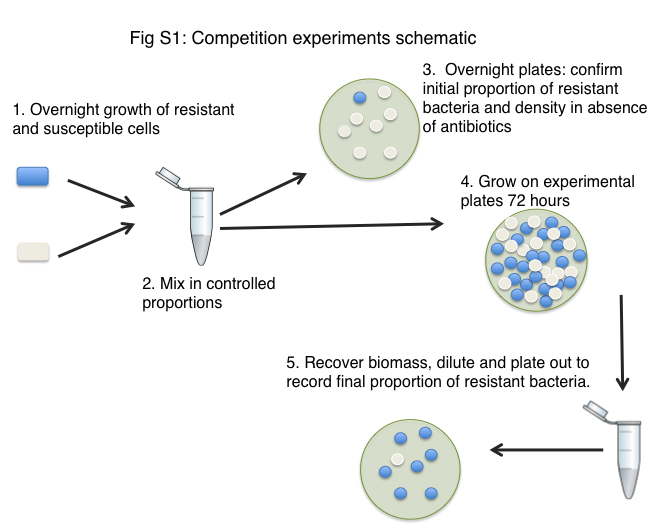


**Supplementary Figure S2. Detoxification of solid media by *E. coli* K-12 MG1655 carrying the pCT plasmid.** Experimental procedures followed those described in the materials and methods except that here we conducted detoxification assays at range of ampicillin doses in the MG1655 background. Note that in the absence of bacteria carrying pCT (top row) the susceptible bacteria were unable to grow at concentrations exceeding the MIC (4 μg ml^-1^). In contrast we when detoxified media for 48 hours with pCT containing bacteria the region of media detoxified sufficiently to allow growth increased with declining dose.


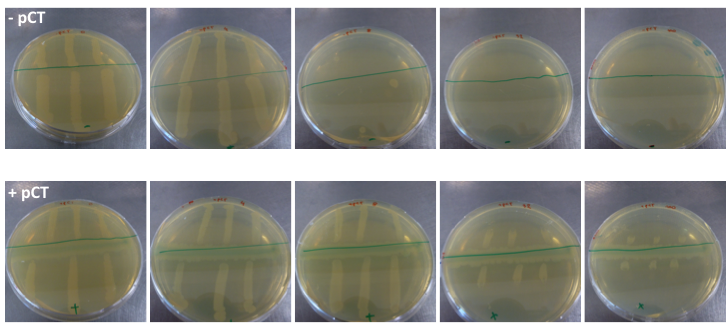


Ampicillin concentration / μg ml^-1^

0 4 8 32 100

**Supplementary Figure S3**. **The relationship between persisters and presence of resistant colonies in assays at 37°C.** Here we repeated experiments described in the main text in figure 3, and grew bacteria for up to 7 days in plates well-wrapped in Parafilm. We see the same phenomenon of late appearing white (genetically susceptible) bacterial satellite colonies growing next to the blue (resistant pCT carrying) colonies at this temperature; photographs were taken after 7 days on plates with 32 (A) or 100 μg ml^-1^ ampicillin (B). We also repeated the results showing a linear relationship between the number of resistant and perister colonies (*F*_1,5_ = 209, *P* = 0.0005) (C) and the increase in persisters with decrease dose of ampicillin (D).


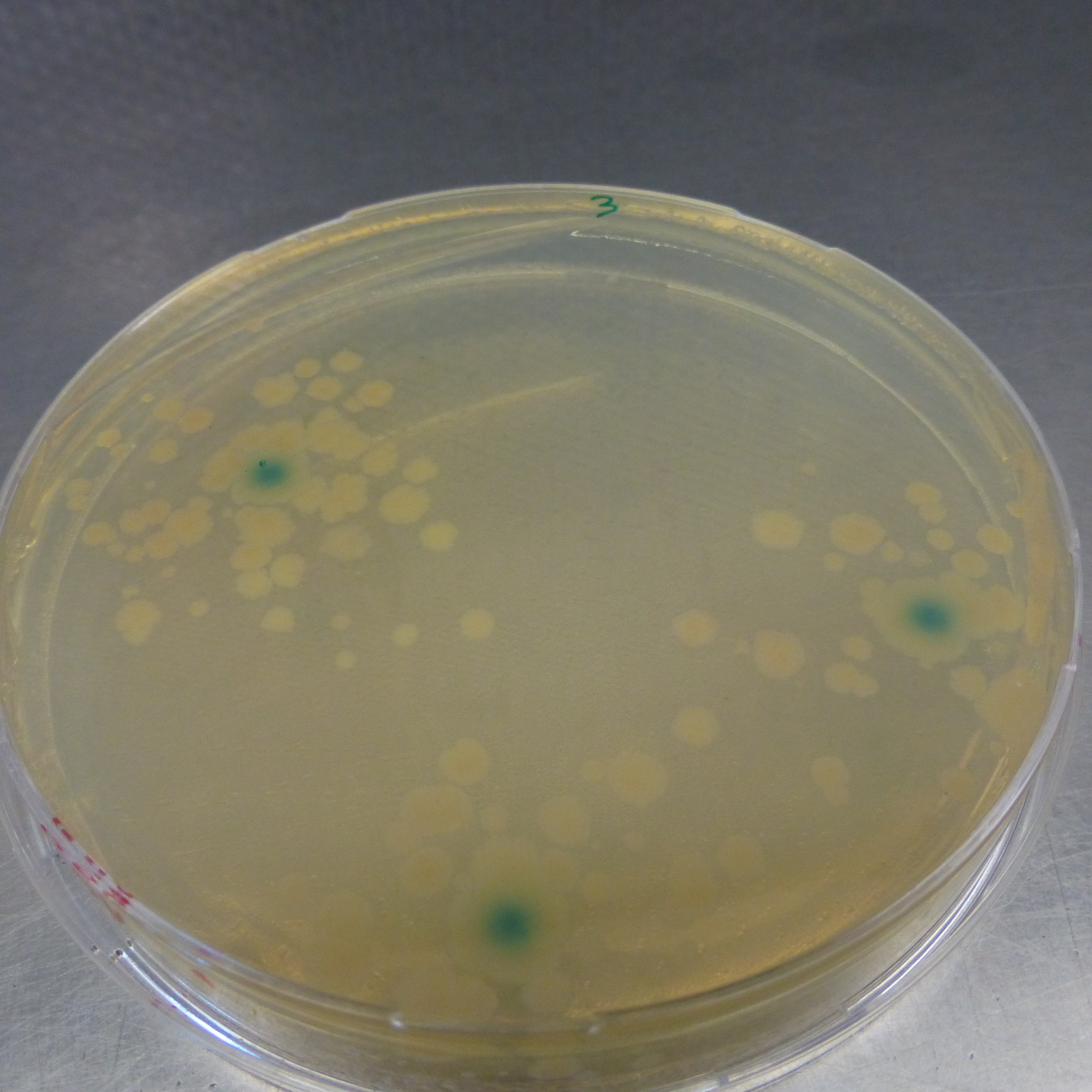

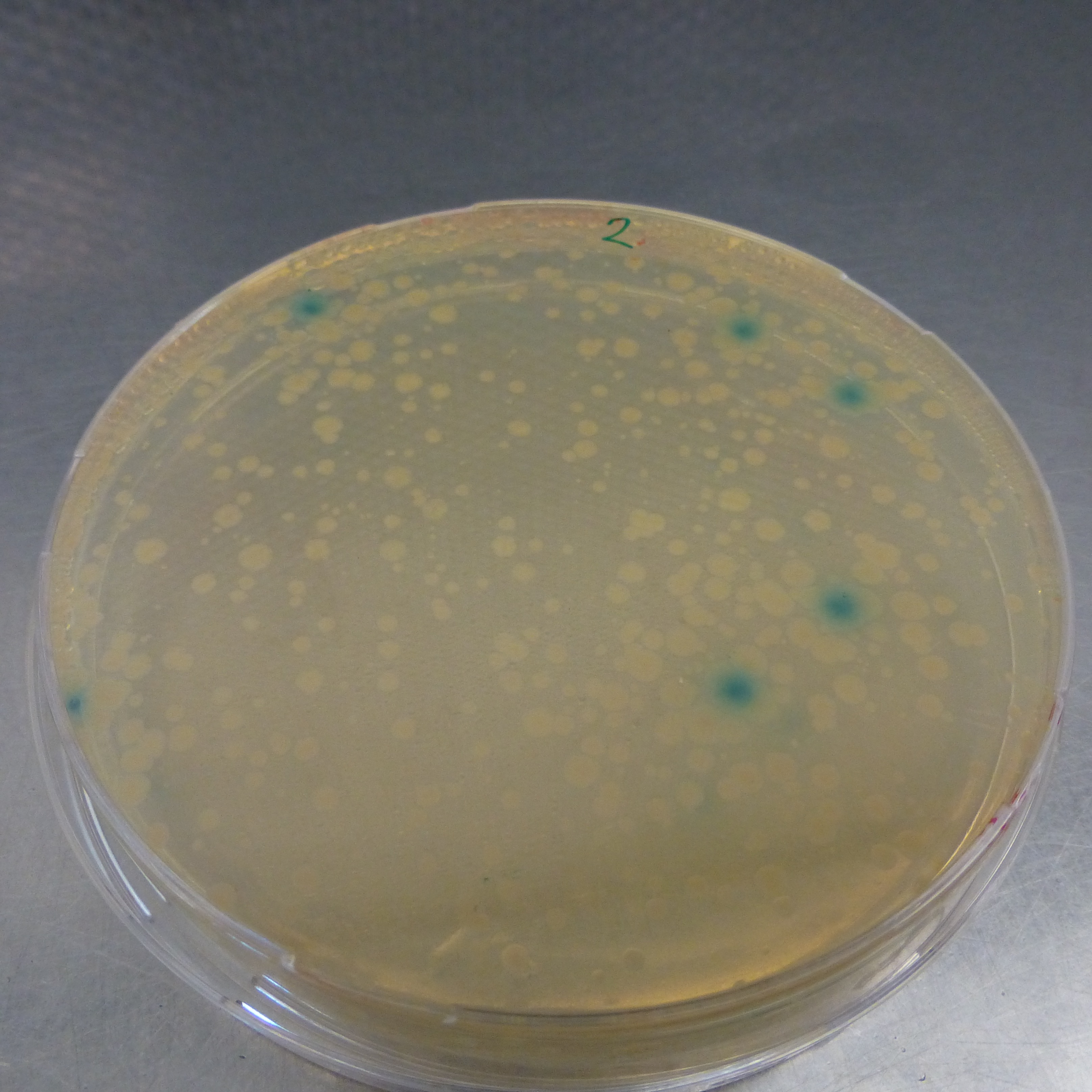


B

A


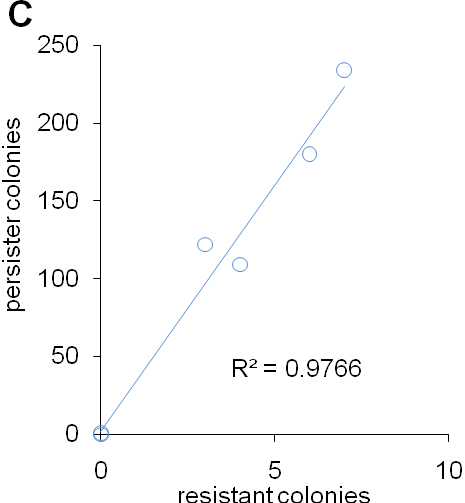

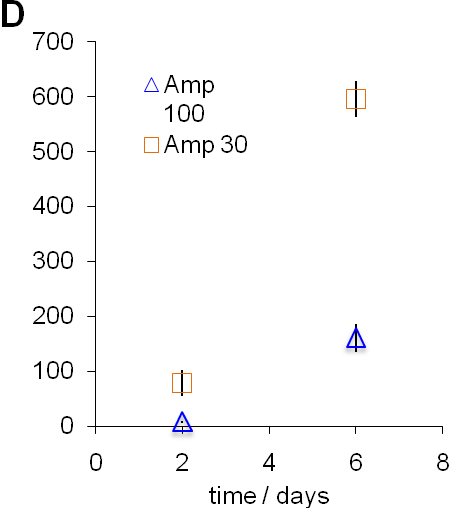

Supplement: Supplementary Information [file ismej2015154x1.doc]
